# Sarcomatoid carcinoma of the urinary bladder: analysis of five cases and literature review

**DOI:** 10.11604/pamj.2020.36.369.25036

**Published:** 2020-08-29

**Authors:** Youssef Kadouri, Salim Ouskri, Hachem El Sayegh, Lounis Benslimane, Yassine Nouini

**Affiliations:** 1 Department of Urology A, Faculty of Medicine and Pharmacy, Ibn Sina Hospital, Mohammed V University, Rabat, Morocco

**Keywords:** Bladder, carcinoma, sarcomatoid, variant

## Abstract

Sarcomatoid carcinomas of the bladder represent a tiny part of bladder tumors and are characterized by a high potential for malignancy. Very aggressive and affecting mainly men, these tumors present both a urothelial and sarcomatoid contingent. The treatment of these tumors is not well codified given the rarity of cases reported in the literature, however, it seems that the treatment is essentially based on radical cystectomy with extensive pelvic lymph node dissection. We report the experience of our departement in the management of this type of tumor in a series of five cases collected over a period of 8 years.

## Introduction

Bladder sarcomatoid carcinomas are a very rare malignant type representing less than 0.5% of bladder tumors, characterized by co-existence of an epithelial urothelial contignent and a spindle cells with sarcomatous connective appearance predominant contingent. It has been considered as an aggressive variant of bladder carcinoma. Less than 100 case reports have been published in the literature. Most cases have been reported as single case reports and small series [1], only one study examining 41 consecutive cases from a single institute exists in the literature [2]. The treatment of sarcomatoid carcinoma of the urinary bladder has not yet been clarified, hence the interest of a multidisciplinary approach in specialized centers.

## Methods

Our work is a retrospective study of medical records, spread over a period of 8 years concerning five cases of sarcomatoid carcinoma of the urinary bladder, diagnosed, treated and followed up in the urology «A » department of the Ibn Sina Hospital in Rabat. By reporting these five cases and an exhaustive review of the literature, using the PubMed database and the guidelines from urology and oncology learned societies, we will assess the epidemiological, clinical, anatomo-pathological and therapeutic characteristics of sarcomatoid carcinoma of the urinary bladder, as well as the study of the evolutionary aspects and prognostic factors.

## Results

**Figure 1:**
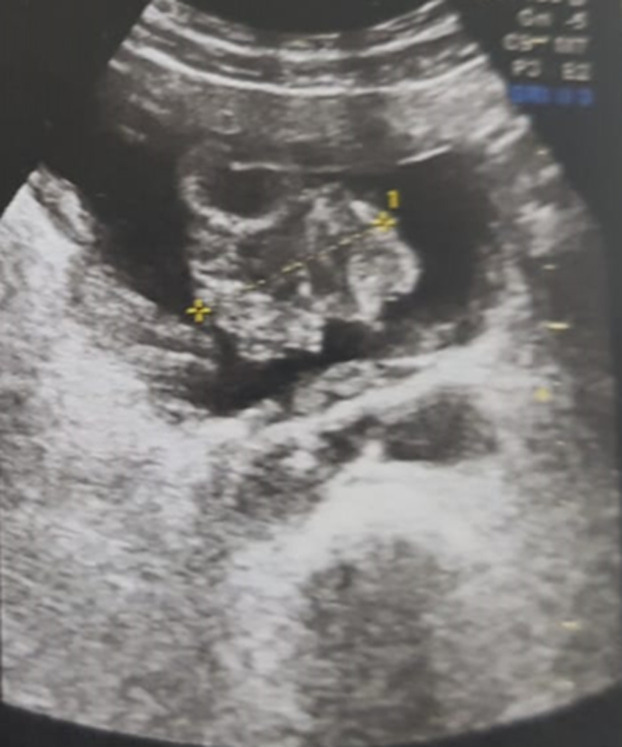
ultrasound showing a parietal mass depending on the anterior surface of the bladder

**Figure 2:**
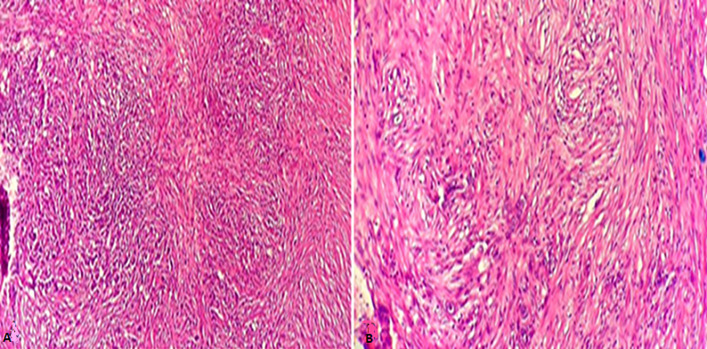
sarcomatoid carcinoma presenting round cells, giant pleomorphic cells and spindle-shaped anisokaryocytic cells. (A) HE original magnification x 100; (B) HE original magnification x200

The characteristics of the patients are detailed in Table 1. They are five men, the average age was 58 years with extremes ranging from 44 years to 70 years. A chronic smoking was found in all of our patients (100%); with an average consumption of 32 PA. The main symptom was a total hematuria with clots, found in all patients, associated with irritant syndrome of the lower urinary tract (pollakiuria and urinary burns) in 4 patients and a general condition deterioration with anorexia and weight loss in two patients. The physical examination was normal in four patients while objectified in the fifth bone pain in the pelvis and the right upper limb. On the paraclinical level: anemia was found in 3 patients, or 60%, with a hemoglobin level ranging from 4g/dl to 12g/dl, secondary to neoplastic disease, as well as to blood spoliation by hematuria. This anemia required a blood transfusion in 3 cases, i.e. in 100% of anemic patients. Kidney failure was found in a patient who improved after performing a percutaneous nephrotomy. On the radiological level: all our patients were examined by a renovesical ultrasound which made it possible to explore the bladder morphology and to suspect the diagnosis of a bladder tumor by objectifying a parietal tissue mass (Figure 1). Transurethral resection of the bladder (TURB) was performed in all of our patients, it was complete in 2 patients and incomplete in 3 because the tumor was endoscopically uncontrollable. It was unique in all patients. An anatomo-pathological examination of the resection chips made the diagnosis for sure in all our patients (Figure 2).

**Table 1:** characteristics of our patients

	Patient N1	Patient N2	Patient N3	Patient N4	Patient N5
Age	44 YO	70 YO	62 YO	67 YO	59 YO
Circumstances of discovery	Total hematuria with clots + irritants syndrome of the lower urinary tract	Total hematuria with clots + irritants syndrome of the lower urinary tract	Total hematuria with clots + irritants syndrome of the lower urinary tract	Total hematuria with clots	Total hematuria with clots + irritants syndrome of the lower urinary tract
Medical background	Chronic smoking	Cholecystectomie chronic smoking	Myocardial infarction whit stent placment 1 year ago; chronic smoking	Appendicectomie chronic smoking cessation 5 years ago	Chronic smoking
Physical examination	Normal	Normal	Normal	Normal	Bone pain in the pelvis and upper right limb
Paraclinical examinations	Ultra sound of the urinary tract : mass of the left lateral wall measuring 3*4cm	Ultra sound of the urinary tract: parietal mass depending on the anterior surface of the bladder	Ultra sound of the urinary tract: huge suspicious parietal mass; anemia	Ultra sound of the urinary tract: huge suspicious parietal mass and pyelo-caliceal cavities dilation; anemia; renal failure	Ultra sound of the urinary tract: huge suspicious parietal mass; anemia
TURB + histological study	TURB was complete and deep; histological study: sarcomatoid carcinoma	TURB was complete and deep; histological study: sarcomatoide carcinoma	TURB was incomplete due to an endosocically uncontrollable tumor; histological study: sarcomatoide carcinoma	TURB was incomplete due to an endosocically uncontrollable tumor; histological study: sarcomatoide carcinoma	TURB was incomplete due to an endosocically uncontrollable tumor; histological study: sarcomatoide carcinoma
Assesment of extention: thoraco-abdomino-pelvic CT Scan	TAP CT scan: localized tumor classified T2N0M0 no secondary localization	TAP CT scan: localized tumor classified T3N0M0 no secondary localization	TAP CT scan: tumor occupying almost the entire baldder locally advanced associated with bilaterale hydro-nephrosis classified T3N2M0 no secondary location	TAP CT scan: locally advanced tumor with pulmonary and lymph nodes metastases	TAP CT scan: locally advanced tumor with pulmonary and lymph nodes metastases; scintigraphy: bone metastases
Treatment	Radical cystoprostatectomie + pelvic lymph node dissection and urinary diversion by enterocystoplastie	Radical cystoprostatectomie + pelvic lymph node dissection and urinary diversion by bricker	Radical cystoprostatectomie + pelvic lymph node dissection and urinary diversion type ureterostomy	Palliative chemotherapy	Refusal of chemotherapy
Immediate postoperative	Simple	Simple	Respiratory distress requiring hospitalization in intensive care for 7 days	N/A	N/A
Long-term follow-up	Favorable with a 2 year follow up	Favorable with a 3 years follow up	Metastatic relapse at 6 months	Died in the 5^th^ month	Patient lost to follow up

**Figure 3:**
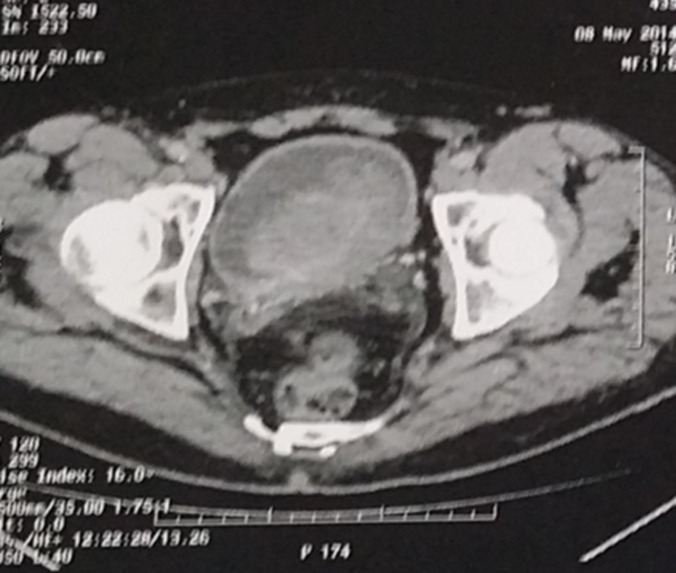
CT scan showing a huge bladder tumor invading the peri-bladder fat classified T3N0M0

The thoraco-abdomino-pelvic CT scann as part of the extension assessment was performed in all our patients and revealed: a localized tumor in 2 patients (classified T2N0M0 in the first and T3N0M0 in the second), a process occupying almost the entire bladder locally advanced associated with bilateral uretero-hydronephrosis classified T3N2M0 in the third patient (Figure 3) and pulmonary and lymph node metastases in the last two patients. Total cystectomy associated with bilateral ilio-obturator lymph node dissection was performed in 3 patients, followed by a urinary diversion by enterocystoplasty in the first patient (T2N0M0), a bricker in the second (T3N0M0) and a cutaneous ureterostomy in the third patient who was operated because of the deterioration of his general condition. Palliative chemotherapy was proposed in the other two patients, however carried out in only one, the second patient had refused treatment. Immediate postoperative follow-up was simple in the first two patients and complicated by respiratory distress in patient number 3 requiring hospitalization in intensive care for 7 days. The long-term evolution was favorable with a 2 to 3 year follow-up in 2 patients or 40% of the cases and unfavorable in 2 patients: metastatic relapse in at 6 months in patient number 3 and death to 5 month in patient number 4. One patient was lost to follow-up.

## Discussion

Sarcomatoid carcinomas of the urinary bladder are very rare malignant tumors, characterized by the co-existence of both an epithelial urothelial contingent and a spindle cells with sarcomatous connective appearance predominant contingent. The carcinomatous contingent is often of high grade. Areas of necrosis are frequently present in the sarcomatoid contingent. Less than 100 cases have been reported in the literature until now [2]. Most cases have been reported as single case reports and small series. It´s a very aggressive type [3], often diagnosed at an advanced stage. It mainly affects men with an average age of 66.4 years [4,5]. Their histogenesis is not clearly identified, however, several pathogenic factors have been reported in the literature such as radiotherapy, smoking and some chemical carcinogens such as cyclophosphamide [6-8]. Clinically, the symptomatology is dominated by hematuria associated or not with irritative signs. The positive diagnosis is based on the anatomo-pathological study of the resection chips. Histologically, the tumor may show a mixture of carcinomatous and sarcomatoid components in varying ratios, but the sarcomatoid component always occupies more than 50% of the tumor area [2]. The epithelial component may be TCC, squamous cell carcinoma, carcinoma in situ, small cell carcinoma and adenocarcinoma, whereas the sarcomatous component could consist of leiomyosarcoma, chondrosarcoma, rhabdomyosarcoma and rarely liposarcoma [5,9]. More than one type of heterologous differentiation may be present [2].

Immunohistochemically, keratin expression was observed focally in the sarcoma component as well as the carcinoma component. Reactivity for vimentin, desmin, muscle specific actin and S-100 protein was observed in poorly differentiated areas in addition to the expected positivity of each histologic subtype of sarcoma [5]. Treatment management remains unclear due to the rarity of the reported cases in literature. Though different treatment modalities have been tried in the literature, yet radical cystectomy followed by adjuvant chemotherapy and radiation should be preferred in all patients, in view of high incidence of local and distant metastasis [10]. This tumor type is effectively radiosensitive [1]. Perret *et al*. underline that patients that survived the longer (up to 12 years) were initially treated by radical cystectomy [5]. Metastatic disease management is based on chemotherapy [11]. The best protocol is still remaining to be defined. The combination of gemcitabine and cisplatin is effective and fairly well tolerated for the treatment of invasive and or metastatic urothelial carcinoma. However, we have no data regarding its use in sarcomatoid carcinomas of the bladder. The use of this chemotherapy protocol in sarcomatoid variants was reported for the first time by Froehner in a single case of sarcomatoid carcinoma in a metastatic (pulmonary) patient and which led to a complete and lasting local and pulmonary remission, nevertheless, no conclusion can therefore be drawn [10].

## Conclusion

Taking into account current series and reviews of the literature, sarcomatoid carcinoma of the bladder is a very aggressive neoplasm occurring mainly in elderly men, diagnosed frequently at an advanced stage and whose evolution is rapidly fatal. Early detection should be used to improve patient outcomes and randomized research should be carried out to improve médical care and establish appropriate treatment guidelines.

### What is known about this topic

- Sarcomatoid carcinoma of the bladder is a very rare tumor;
- Its therapeutic management is poorly codified given the rarity of cases reported in the literature.

### What this study adds

- The diagnosis of sarcomatoid carcinoma should be evoked even in the absence of known risk factors for this pathology;
- Our study confirms the place of total cystectomy with lymph node dissection held as standard treatment for this rare tumor;
- Enterocystoplasty remains indicated as a method of urinary diversion in patients in good general condition, managed quickly.
